# The Influence of Prophylactic Calcium and Magnesium Supplementation on Postoperative Quality of Life and Hypocalcemia After Total Thyroidectomy: Study Protocol for a Randomized Controlled Trial

**DOI:** 10.3389/fsurg.2021.758205

**Published:** 2022-01-06

**Authors:** Navid Tabriz, Dennis Fried, Verena Uslar, Dirk Weyhe

**Affiliations:** University Hospital for Visceral Surgery, Carl von Ossietzky University Oldenburg, Oldenburg, Germany

**Keywords:** preoperative calcium supplementation, preoperative magnesium supplementation, thyroidectomy, postoperative hypocalcemia, quality of life, ThyPRO-39, EQ-5D-5L

## Abstract

**Background:** We want to investigate if a routine preoperative dietary supplementation of calcium and magnesium prior to thyroidectomy for nodular goiter and graves' disease can influence patients' outcome with regards to hypocalcemia associated symptoms and quality of life in order to reduce the risk of post-thyroidectomy hypocalcemia and to improve patient's quality of life.

**Methods:** The study will be conducted as a two-armed randomized controlled trial including patients scheduled for total thyroidectomy. Patients assigned to the intervention group will receive calcium carbonate and magnesium oxide starting 2 weeks preoperatively. Primary outcome is the postoperative quality of life measured by the ThyPRO-39 and EQ-5D questionnaires. Secondary outcome is the assessment of postoperative biochemical (calcium and PTH levels) and clinical hypocalcemia (symptoms as reported by the patient).

**Discussion:** A prophylactic dietary supplementation with calcium and magnesium, which could easily be implemented in the preoperative setting, could potentially help to avoid or reduce hypocalcemia-associated symptoms and improve quality of life. In the event of a positive outcome, this preoperative procedure can be an inexpensive way to prepare patients scheduled for thyroidectomy and can possibly reduce disease-specific costs by reducing the postoperative complication rate.

**Clinical Trial Registration:** DRKS00017195 in the German clinical trials register (Deutsches Register Klinischer Studien, DRKS) on the 22.05.2019.

## Introduction

Thyroid surgery is one of the most common surgeries worldwide (1). Although the total number of thyroid surgeries has decreased in Germany in the last years, the proportion of total thyroidectomies (TT) is increasing (2). Total thyroidectomy is performed for example in case of malignancy, suspected bilobular or symptomatic nodular goiter or graves' disease. Hypocalcemia is one of the most common complications of bilateral thyroid resections with an incidence ranging from 0.3 to 49% (3). Symptoms of postoperative hypocalcemia can range from mild paresthesia and peripheral muscular spasms to laryngospasm and life-threatening cardiac arrhythmias (4). These symptoms do not only lead to extended hospital stay but also reduces patients' quality of life (5, 6). Therefore, serum calcium (and) parathyroid hormone (PTH) levels are monitored in the early postoperative course. Especially, due to the very short half-life of PTH (3–5 mins), early postoperative PTH determination can help to detect postoperative hypoparathyroidism. PTH levels <15 pg/mL at least 20 mins or more following TT might predict potential therapy-relevant hypoparathyroidism (7, 8). In a recent study, a cut-off PTH value of >19.95 pg/mL during the immediate postoperative period was established for a safe early discharge without supplementation of calcium and vitamin D (9). Despite this clinically accepted postoperative procedure, there are no generally accepted postoperative hypoparathyroidism management recommendations. Recently, a meta-analysis has confirmed the effectiveness of calcium and vitamin D in the postoperative course for preventing hypocalcemia-associated symptoms. Yet, a routine supplementation could also lead to overtreatment in patients with normal serum calcium (10). In one of the few studies dealing with preventive preoperative calcium supplementation prior to total thyroidectomy it was shown that preoperative calcium carbonate supplementation effectively reduced postoperative biochemical and symptomatic hypocalcemia (11).

Evidence exist that thyroidectomy can cause significant hypomagnesaemia, possibly correlating with the development of hypocalcemia (1, 12–14). Therefore, postoperative monitoring after thyroidectomy, should include controlling both, serum calcium and magnesium.

This background provides the motivation for this study, which aims to investigate the quality of life and the development of clinical hypocalcemia in relation to preoperative calcium and magnesium administration. We want to investigate whether a routine preoperative dietary supplementation of calcium and magnesium influences patients' outcome with regard to hypocalcemia-associated symptoms and quality of life in order to reduce the risk of post-thyroidectomy hypocalcemia and to improve patient's quality of life. This could potentially reduce hospital length of stay and medical expenses.

The primary aim of this study is to investigate the influence of preoperative calcium and magnesium supplementations starting 2 weeks prior to surgery on quality of life measured by the ThyPRO-39 questionnaire over a time interval of 2 weeks prior to 6 weeks post-surgery in a randomized controlled trial.

The secondary aim is to learn how the aforementioned supplementation may influence the development of clinical and biochemical hypocalcemia. In addition, the ThyPRO-39 and the EQ-5D-5L questionnaires outcomes should be compared at the time of hospital discharge.

## Methods

### Trial Design

**Figure 1:**
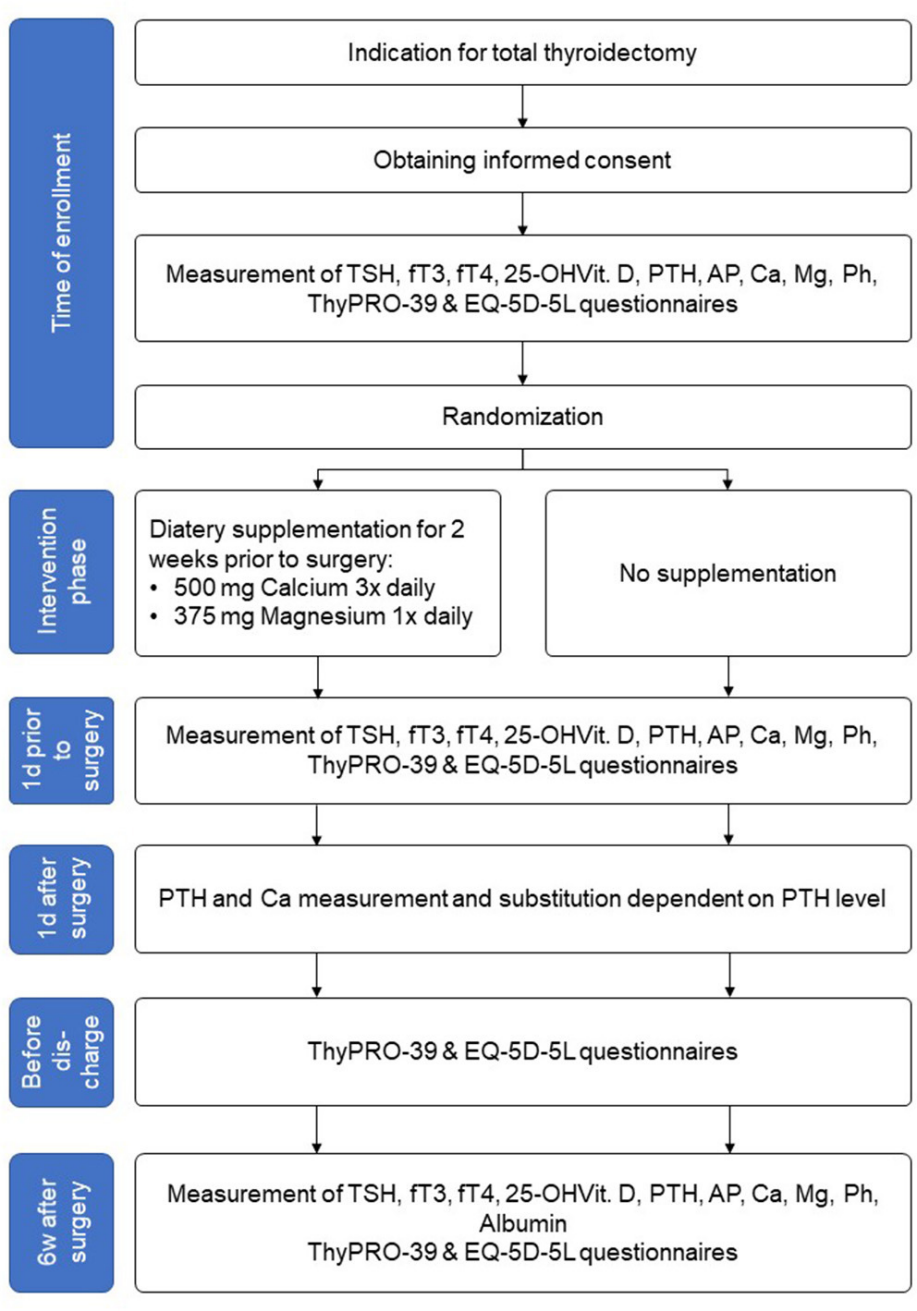
Study flow chart.

We propose a prospective randomized controlled intervention study with a postoperative follow-up of 6 weeks (Figure 1; Table 1).

**Table 1:** Schedule of enrolment, interventions, and assessments according to SPIRIT statement.

	**Study period**						
	**Enrolment**	**Post-allocation**					**Close-out**
**TIMEPOINT**	***Indication for surgery***	***2w prior to surg***	***1w prior to surg***	***1d prior to surg***	***1 d after surg***	***Before dis-charge***	***6w after surg***
**Enrolment:**							
Eligibility screen	X						
Informed consent	X						
Allocation	X						
**Interventions:**							
Mg and Ca supplementation^*^		X	X	X	X	X	X
*No supplementation*		X	X	X	X	X	X
**Assessments:**							
*TSH, fT3, fT4, 25-OHVit. D, AP, Mg, Ph, Albumin*	X			X			X
*PTH & Calcium*	X			X	X		X
*ThyPRO-39 &* *EQ-5D-5L questionnaires*	X			X		X	X
*Age, weight, size and results of the ultrasound thyroid exam*	X						X
*Phonecall about well-being and side effects*			X				

* *500 mg Calcium 3x daily & 375 mg Magnesium 1x daily*.

### Study Setting

The monocentric study will be conducted at a DGAV (Deutsche Gesellschaft für Allgemein- und Viszeralchirurgie; German Society for General and Visceral Surgery) certified center for thyroid and parathyroid surgery in a German university hospital for visceral surgery. The hospital performs more than 350 thyroid surgeries per year, of which about 100 are TTs.

### Eligibility Criteria

Inclusion criteria:

- Adult patients (> 18 years)
- Scheduled for TT
- Diagnosis: symptomatic bilobular nodular goiter or graves' disease
- Thyroid cancer treated with total thyroidectomy without lymph node dissection

Exclusion criteria:

- Lack of written consent
- Thyroid cancer treated with TT and lymph node dissection
- Mediastinal goiter with need for sternotomy
- Inability to communicate in German
- Medication with thiazide diuretics, digitalis or lithium therapy
- Previous neck operations or radiation
- Preexisting hyperparathyroidism
- Chronic kidney failure.

Drop out criteria:

- Withdrawal of consent
- Inability to follow the supplementation regimen
- Necessity of following central or lateral neck dissection due to malignancy
- Accidentally removed parathyroid gland or autologous reimplantation of removed parathyroid gland

### Informed Consent and Disenrolment

Informed consent will be obtained prior to patient enrolment. The consent form will be validated using both the physicians' (DF or, in absence, NT) and patients' signatures.

Participation will be voluntary, and patients may quit the trial at any time without disclosing their motives. Disenrollment will not affect subsequent medical care. In the case of withdrawal, relevant data will be deleted if desired by the patient. Patients' names and all other confidential information are patient of medical confidentiality under the German Data Protection Act. Any data transmission will be performed using an encrypted format. Third parties not involved in the trial will not have access to original documents. This study will be carried out in accordance with the Helsinki Declaration in its current version. The local university's medical ethical committee has approved the study protocol (No. 2017-105).

## Interventions

### Intervention Description

One group (intervention group; IG) will receive calcium carbonate and magnesium oxide nutritional supplementation. Ingestion will begin 2 weeks preoperatively. The comparison group (control group; CG) will receive no nutritional supplements. The chosen supplementation period allows to build-up significantly higher calcium and magnesium levels in IG-patients compared to CG-patients.

Patients assigned to the IG will receive calcium carbonate 3 × 500 mg/d (Calcium Sandoz^®^ 500 mg, Hexal) and magnesium oxide 1 × 375 mg/d (Magnetrans^®^ 375 mg, StadaVital).

### Criteria for Discontinuing or Modifying Allocated Interventions

One week after having started supplementation, IG-patients will be contacted *via* phone to evaluate their well-being and ask for any side effects. If patients report any intervention-related side effects (e.g., diarrhea), patients may reduce the dose or stop ingestion. Those patients will be excluded from the analysis.

### Strategies to Improve Adherence to Interventions

The planned phone call is meant to improve adherence.

### Relevant Concomitant Care Permitted or Prohibited During the Trial

Not applicable.

### Provisions for Post-trial Care

Since the supplementation dosage is within the range of recommended calcium and magnesium uptake, no harm from trial participation is expected. However, patients are encouraged to contact the involved physicians at any time.

### Outcomes

Primary outcome will be the postoperative quality of life measured by the ThyPRO-39 questionnaire over time between both groups.

Secondary outcomes will be the post-operative biochemical levels of calcium and PTH, the development of clinical hypocalcemia (symptoms as reported by the patient) during post-operative hospital stay, and the ThyPRO-39 and EQ-5D-5L results at hospital discharge.

### Participant Timeline

After checking the indication for total thyroidectomy and obtaining informed consent, the patients's quality of life will be assessed by the health-related EQ-5D-5L and the disease-specific questionnaire ThyPRO-39 (Figure 1). Laboratory tests for thyrotropin (TSH), free triiodothyronine (fT3), free tetraiodothyronine (fT4), calcium, magnesium, phosphate, albumin, 25-OH vitamin D3, PTH, alkaline phosphatase (AP) will be performed. Patients' biometric data (age, weight, size, body mass index) and the ultrasound thyroid examination results will be recorded.

Patients assigned to the intervention group will start supplementation as described above.

One day before the scheduled operation, the ThyPRO-39 questionnaire will be filled out again and PTH, calcium and magnesium levels will be determined.

Thyroidectomy will be, by default, performed under intraoperative neuromonitoring of the recurrent laryngeal nerves. Intraoperatively, the macroscopic view of the parathyroid glands and description of structure and blood flow are required. If the lower parathyroid glands are not in loco typico, an explicit representation is not necessary. In the case of an accidentally removed parathyroid gland or insufficient blood supply, the gland is removed, cut into small cubes and autologously reimplanted in a muscle pocket of the sternocleidomastoid muscle. The affected patient is then excluded from the study.

Postoperatively, all patients are routinely monitored on the intermediate care unit, especially with regard to potential postoperative hemorrhage. PTH and calcium will be checked on first postoperative day. Depending to the measured PTH level (norm 15–65 pg/ml) calcium with or without Alfacalcidol (Einsalpha^®^, LEO) will be supplemented, regardless of group affiliation (Table 2).

**Table 2:** Postoperative procedure dependent on PTH levels on first postoperative day.

	**PTH <15 pg/ml**	**PTH 15-30 pg/ml**	**PTH 30–65 pg/ml**	**PTH > 65 pg/ml**
Alphacalcidol	0,5 μg 2 × d	–	–	–
Calcium carbonate	2 × 1 g	2 × 1 g	as required 1 g	as required 1g
Magnesium oxide	2 × 375 mg	375 mg	–	–

Before patient's discharge, the EQ-5D-5L and ThyPRO-39 will be completed for a third time. Patient's discharge is planned on the 2nd or 3rd postoperative day depending on his condition. The histological result will be available within 24–48 h postoperatively. In case of benign histology, L-Thyroxin substitution is started at a dosage of 1.5 μg per body weight and is adjusted at the following examination after 6 weeks depending on thyroid parameters.

Six weeks postoperatively laboratory examinations for TSH, fT3, fT4, calcium, magnesium, phosphate, albumin, 25-OH vitamin D3, PTH, AP and ThyPRO-39 questionnaire will be carried out during a clinical visit. In case of PTH <15 pg/ml, the therapeutic goal of vitamin D-, calcium- and magnesium-substitution is to achieve a serum calcium level in the lower normal range (2.0–2.2 mmol/l). At the beginning of the substitution, calcium and phosphate values should be determined at 2-weekly intervals and vitamin D dose should be adjusted by 10–20% depending on the calcium level. The laboratory exams will be repeated after 6 months to determine a potential persistent hypoparathyroidism.

### Sample Size

We aim for a patient population of 80 male and female adults. This is based on a sample size calculation using data from our own validation study of the ThyPRO-39de (15). For sample size calculation the free software G^*^Power (Version 3.1.9.4) was used. The sample size calculation for the between-factors (i.e., supplementation or not) Repeated Measures Analysis of Variance (RM ANOVA) for the test of the primary outcome was based on the following assumptions:

- Effect size f = 0.25
- α err prob = 0.05
- Power (1-β err prob) = 0.8
- Number of groups = 2
- Number of measurements = 4
- Correlation among repeated measures = 0.4

The effect size was calculated based on a mean of 30 points and a standard deviation of 19.6 for the ThyPRO-39 composite score for the control group [according to (15)]. We assumed a mean decrease of about 10 points, which reflects a clinically relevant change, and no change in standard deviation for the intervention group. This yields the aforementioned effect size. Using these assumptions, an overall sample size of 72 patients is needed. To account for drop outs, we are planning to include 80 patients.

### Recruitment

All patients scheduled for total thyroidectomy at our hospital will be asked to participate in this trial by one of the responsible physicians in case indication for surgery and eligibility criteria are met. After explaining the study procedures to eligible patients in an accessible way, patients are asked for written consent. Since about 100 patients are surgically treated by total thyroidectomy in our hospital per year, we expect to enroll 80 patients in about 18–24 months. The recruitment period will be extended if, for some unforeseen reason, the target sample size is not met within this period.

## Assignment of Interventions: Allocation and Blinding

### Sequence Generation

A block randomization with a block length of 6 will be applied. The randomization script is written in Matlab by an uninvolved researcher (VU).

### Concealment Mechanism

Sealed consecutively numbered envelopes will be prepared, where each envelope will contain a slip of paper indicating group allocation.

### Implementation

The allocation sequence will be generated by VU as previously reported. All other personnel will be blinded to the exact randomization method used. The same uninvolved researcher will also prepare the sealed envelopes. The envelopes will be stored in the study office. Patients will be enrolled either by DF or NT. After patients have provided their written informed consent, the next envelope will be opened by the investigator or an assigned study nurse in front of the patient, and will thus assign participants to the intervention or control groups as described above.

### Who Will Be Blinded

The surgeons will know about any patient's study enrollment but will be unaware of patients' group allocation. The physicians and study nurses mentioned as authors are responsible for data collection and need to be aware of the treatment arm in order to assess any side effects.

### Procedure for Unblinding if Needed

Not applicable.

## Data Collection and Management

### Plans for Assessment and Collection of Outcomes

The physicians and study assistants responsible for data collection will collect all data on paper and in an Excel spreadsheet.

### Plans to Promote Participant Retention and Complete Follow-Up

As all important outcome variables will be collected during hospital stay, it is expected that there will be an almost complete follow-up. This will be additionally ensured by the study team informing themselves daily about any patient's time of discharge, and contacting corresponding patients in time to obtain any missing data. In case a patient wants to terminate study participation prior to the final follow-up appointment, they will be asked whether their so-far collected data may still be used.

### Data Management

Data will be collected either on paper (i.e., the questionnaires, conversations with the patient) or by obtaining relevant data from the hospitals' patient database (e.g., laboratory values, complications). Paper-based information will be kept in a secure, locked cabinet, which only authorized study personnel can excess. All data will be entered into a pseudonymized excel spreadsheet by only two different individuals (DF and the responsible study nurse). The spreadsheet will be kept on a hospital computer to which only authorized study personnel has access. A codebook will be available in the spreadsheet to ensure correct data entry. The data will be checked for plausibility and completeness by an independent third person (VU). A list including patients' names and contact information connecting each patient to their pseudonym will be kept in a separate excel spreadsheet, which can only be accessed by DF and the responsible study nurse, such that it can be destroyed after data curation.

### Confidentiality

All documents will be accessible only for study personnel. Electronic documents will be password protected. A coding list will be created in which patients' names will be entered following their assent to participate. This coding list will contain each patient's real name and contact details, as well as a unique random study number. The aforementioned pseudonymized Excel spreadsheet only contains the corresponding study number. After data collection is completed, the coding list will be deleted, so that only the Excel spreadsheet with completely deidentified data is available. This password secured Excel spreadsheet will be stored for 10 years in accordance with the recommendations for good clinical and scientific practice, and will be available upon request to other researchers. After this period, this list will also be deleted.

### Plans for Collection, Laboratory Evaluation and Storage of Blood Samples

Laboratory evaluation will be based on blood samples, which are routinely collected during the hospital stay and follow-up appointments. As such, all data will be collected in the regular clinical database and extracted using patients' pseudonyms generated for this study.

## Statistical Methods

### Statistical Methods for Primary and Secondary Outcomes

EQ-5D-5L- and ThyPRO-39 will be analyzed according to previous recommendations. Patient characteristics, QoL questionnaire scores, and laboratory findings will be analyzed descriptively, separately by intervention and by follow-up time. Means or proportions will be reported as appropriate, and accompanied by 95% confidence intervals. Interferential statistics will be performed using a between-factors repeated-measures ANOVA (i.e., supplementation or not) for the primary outcome QoL over time as measured with the ThyPRO-39. The chi-square test or Fishers exact test will be used for between-group difference calculations regarding the development of postoperative clinical hypocalcemia. For comparison of the EQ-5D-5L questionnaire with the ThyPRO-39 at the time of discharge, the *t*-test will be used. A *p-*value < 0.05 is used as a measure of statistical significance. For all statistical tests, the respective effect size (?^2^) will be reported.

### Interim Analyses

No Interim Analysis Is Planned.

### Methods for Additional Analyses (e.g., Subgroup Analyses)

As the pathomechanism of nodular goiter and graves' disease differ, a subgroup analysis for these two diseases will be performed. Patient characteristics, QoL questionnaires results, and the results of laboratory evaluations will be analyzed descriptively within both groups for both diseases separately, reporting means or proportions as appropriate, together with the 95% confidence intervals.

### Methods to Handle Protocol Non-adherence and Missing Data

Patients allocated to the intervention group but for whatever reason not undergoing the intervention are excluded from the analysis. Missing values will be handled using multiple imputation.

### Plans to Give Access to the full Protocol, Participant Level-Data and Statistical Code

All data will be made available upon reasonable request from the corresponding author.

## Oversight and Monitoring

### Composition of the Coordinating Center and Trial Steering Committee

The trial steering committee will be comprised of NT, DF and the study nurse. They will conduct all measurements and report to the data monitoring committee. In addition, DF is responsible for data collection and data entry.

### Composition of the Data Monitoring Committee, Its Role and Reporting Structure

VU and DW constitute the data monitoring committee. VU will be responsible for checking data completeness and plausibility, while DW functions as the sponsor for this study. The study is initiated by the involved scientists. There are no competing interests, although an amount of 3000*$* was provided by Handke Medizintechnik GmbH for additional laboratory examinations in the follow-up period. Handke Medizintechnik GmbH is no member of any committee, and does not have a say neither with regards to the trial design nor to the reporting of the results.

### Adverse Event Reporting and Harms

Any occurring adverse event will be reported to the medical ethic committee of the Carl von Ossietzky University Oldenburg.

### Frequency of and Plans for Auditing Trial Conduct

After the first 5 patients have been enrolled, the implementation procedure will be reviewed by the trial steering committee, together with the data monitoring committee to ensure that the procedure is appropriate. In case of arise problems, appropriate steps will be taken to assure that the protocol will be followed, or, if needed will be changed accordingly. Following, a monthly auditing will be implemented to keep all personnel up to date. The auditing will take place in form of discussions with the entire study team. Further meetings will be held if necessary.

### Plans for Communicating Important Protocol Amendments

If the audits reveal that changes to the study protocol are mandatory, appropriate amendments will be submitted to the responsible ethics committee. Moreover, such changes to the protocol will be reported to the DRKS trial registration system. Participants will be informed if necessary.

## Dissemination Plans

Results will be published in a medical journal.

## Discussion

Hypocalcemia following total thyroidectomy implies potential impairment of the parathyroid glands, which can negatively impact patients' treatment outcomes, prolongs hospital stay and increases treatment cost. Currently, in case of total thyroidectomy serum calcium concentration and PTH levels are monitored postoperatively followed by needs-based calcium and vitamin D supplementation. Through this regime, transient hypocalcemia can be adequately treated in most cases. PTH can already been determined 20 mins after TT and cut-off values of <15 pg/mL or <20 pg/mL, also depending on the assay used, can help to predict the likelihood of therapy-relevant postoperative hypoparathyroidism (7–9). Due to clinical practical application, we decide to determine PTH and calcium level on the first postoperative day and to substitute calcium with or without vitamin D depending on the PTH value. Thereby, supplementation of calcium and vitamin D can be applied to facilitate a safe early discharge leading to cost savings, for instance by reducing inpatient treatment time, and improved patients' satisfaction and quality of life.

There is ample evidence, including meta-analysis and systematic reviews, confirming the effectiveness of early postoperative calcium and vitamin D supplementation on postoperative outcome after TT (10, 16). Yet, only few studies have examined the role of a prophylactic preoperative calcium (and) vitamin D supplementation on postoperative hypocalcemia. In a prospective study with pre- and postoperative calcium and vitamin D supplementation in 50 patients undergoing TT, the incidence of symptomatic hypocalcemia was 6% and of laboratory hypocalcemia it was 10%. However, that study did not include a no control group (17). Jaan et al. (18) examined 46 patients who underwent total or near TT in a randomized controlled trial. They randomly divided the patients into two groups, one group (*n* = 24) receiving calcium and vitamin D 7 days pre- and 7 days postoperatively, and one group did not receive such supplementation. They concluded that routine vitamin D and calcium supplementation can significantly reduce postoperative hypocalcemia. However, in that study perioperative PTH levels and vitamin D deficiency were not measured and different thyroid diseases (thyroid cancer, graves' disease and nodular goiter) were included. In graves' disease, it is well known that patients have much higher rates of symptomatic hypocalcemia after total thyroidectomy than with other diagnosis due to, for example, the “hungry bone syndrome” and blood supply disturbances of the parathyroid glands (19–21). Oltmann et al. (11) examined the effect of 2 weeks of preoperative calcium supplementation (3 grams/day) on postoperative hypocalcaemia in comparison to a historic matched non-supplemented group with graves' disease. They stated that calcium supplementation before TT for graves' disease significantly reduces biochemical and symptomatic postoperative hypocalcemia.

It has to be pointed out that for comparison of different thyroid diseases such as graves' disease, nodular goiter, or cancer, subgroup analysis should be performed as the pathophysiology of postoperative hypoparathyroidism depends on the primary diagnosis and on the extent of surgery (i.e., TT plus lymph node dissection). In order to avoid surgery-related hypoparathyroidism due to central or lateral lymph node dissection, only TT for benign diseases such as symptomatic nodular goiter or graves' disease is included in our trial.

Studies on the role of preoperative vitamin D level on early postoperative hypocalcemia reveal contradictory results. On the one hand, low preoperative vitamin D and low postoperative parathyroid hormone levels were seen as predictors for the development of hypocalcemia (22, 23). On the other hand, these biochemical parameters could not be detected as a risk factor in other studies (9, 24, 25). Therefore, for our planned study, the influence of vitamin D level on postoperative hypocalcemia is also regarded.

We assume that a prophylactic dietary supplementation with calcium and magnesium, which could easily be implemented in the preoperative setting, could potentially help to avoid or reduce hypocalcemia-associated symptoms and improve quality of life. Due to lacking data concerning the optimal duration for a preoperative supplementation period, we have pragmatically used the data of Oltmann et al. (11). Also, the amount of calcium and magnesium to be administered could be subject to discussion. We decided to prescribe 1500mg calcium and 375 mg magnesium that is not harmful for patients, as potential side effects are rare and associated with much higher doses, according to the EFSA-recommendations (26, 27).

Following the here presented preoperative procedure may be useful to prepare patients scheduled for thyroidectomy and can possibly reduce disease-specific costs and improve patient's quality of life.

## Trial Status

This protocol corresponds to version 3.0 of the study design, which has been positively reviewed by the Medical Ethics Committee (No. 2017-105). Recruitment started on 19.03.2019 and will probably be completed in late in 2022. Currently 35 of the targeted 80 patients are recruited. The trial was registered with the DRKS (Deutsches Register Klinischer Studien; German Clinical Trials Registry) on 22.05.2019.

## Ethics Statement

The studies involving human participants were reviewed and approved by Medical Ethics Committee of the Carl von Ossietzky Universty Oldenburg /Ref No. 2017-105. The patients/participants provided their written informed consent to participate in this study.

## Author Contributions

NT is the principal investigator and developed the study design and wrote the first draft of the manuscript. DF will be mainly responsible for data collection and contributed to the study design, the ethics proposal, and revision of this manuscript. VU was responsible for the scientific evaluation of the study design, development of the statistic analysis plan, and wrote the respective parts of this manuscript. DW is sponsor of this study and contributed to the study design and revised the manuscript. All authors contributed to the article and approved the submitted version.

## Funding

Funding was received in the form of financial support (3,000*$*) from Handke Medizintechnik GmbH. The funder was not involved in the study design, collection, analysis, interpretation of data, the writing of this article or the decision to submit it for publication.

## Conflict of Interest

The authors declare that the research was conducted in the absence of any commercial or financial relationships that could be construed as a potential conflict of interest.

## Publisher's Note

All claims expressed in this article are solely those of the authors and do not necessarily represent those of their affiliated organizations, or those of the publisher, the editors and the reviewers. Any product that may be evaluated in this article, or claim that may be made by its manufacturer, is not guaranteed or endorsed by the publisher.
